# Eye Injuries Epidemiology Description in a Working Population over 10 Years in Spain

**DOI:** 10.3390/ijerph17124454

**Published:** 2020-06-21

**Authors:** Sergio Martín-Prieto, Cristina Álvarez-Peregrina, Israel Thuissard-Vassallo, Carlos Catalina-Romero, Eva Calvo-Bonacho, César Villa-Collar, Miguel Ángel Sánchez-Tena

**Affiliations:** 1 School of Biomedical and Health Science, Universidad Europea de Madrid, 28670 Madrid, Spain; cristina.alvarez@universidadeuropea.es (C.Á.-P.); israeljohn.thuissard@universidadeuropea.es (I.T.-V.); villacollarc@gmail.com (C.V.-C.); miguelangel.sanchez@universidadeuropea.es (M.Á.S.-T.); 2 Ibermutua (Mutual Collaborator of Social Security nº 274), 28043 Madrid, Spain; carloscatalina@ibermutua.es (C.C.-R.); evacalvo@ibermutua.es (E.C.-B.)

**Keywords:** eye injuries, eye protective devices, occupational injuries, epidemiology, injury epidemiology, injury prevention

## Abstract

Background: Several studies show a high percentage of eye injuries related to work compared to other origins. However, there are few studies that describe work-related eye injuries. Methods: A descriptive, retrospective, and longitudinal study that describes the characteristics of work-related eye injuries in a group of insured workers. Eye injuries were classified according to the International Classification of Diseases (ICD-10) and analyzed over 10 years (2008–2018). Results: Keratitis and conjunctivitis were the most prevalent injuries (26,674 (53.1%) and 15,906 (31.6%)). Keratitis and conjunctivitis also show the highest percentage of injury incidence per 100,000 insured workers in both sexes, any age group, and any occupation. The analysis of the cumulative percentage change and average annual percent change in incidences over ten years shows a decrease in the incidences of all injuries, except for other disorders of the eye and anexa. Conclusions: Most of the work-related eye injuries affect the most exposed eye structures in any line of work: the cornea and conjunctiva. Suitable protection of these eye structures will decrease the number of cases of work-related eye injuries.

## 1. Introduction

The percentage of patients with ocular injuries, suffered while working, that receive urgent care from hospitals varies from 5.6% to 56.5% [1,2,3]. In Spain, two studies were conducted in 1994 and 2008, which saw a percentage of patients with ocular injuries that went from 23.0% to 25.2% [4,5]. In 2018 in Spain, 17,579 workers were on sick leave due to a work-related eye injury [6].

According to the scientific literature, the relative risk of suffering a work-related eye injury (WREI) is higher in men, younger and less experienced workers, and in those who do manual work [1,2,7,8].

A high percentage of eye injuries are produced by fragments or foreign bodies and affect the anterior segment of the eyeball [1,7,8,9,10]. In a study published by Fea et al. in Torino (Italy), 54.22% of WREIs were conjunctivitis and corneal injuries [3]. Several studies have proved that 70% of workers that suffered an eye injury did not wear eye protection [2,11].

Nowadays, there are few studies across Europe to describe the most prevalent types of work-related eye injury. The main objective of this study is to describe the most prevalent WREIs in Spain during the last 10 years and analyze the main risk factors. An accurate description of the most prevalent injuries at work and about which part of the eyeball is affected should help companies and governments to make special policies to prevent WREIs.

## 2. Materials and Methods

### 2.1. Study Design

A descriptive, retrospective, and longitudinal study was performed. The study data were provided by Ibermutua, a mutual insurance company that collaborates with the Spanish Social Security system. In this company, medical specialists evaluate work-related injuries reported by the companies they insure, analyzing the work-related injuries and their consequences for insured workers.

The study period was from 1st January 2008 to 31st December 2018. All the work-related injuries were analyzed and compared with the number of insured workers every year over 10 years.

The research described herein adhered to the tenets of the Declaration of Helsinki and approved by the ethics investigation committee of Universidad Europea de Madrid (CEI-UE). All medical records were anonymous; only statistical information was provided by Ibermutua for research purposes.

### 2.2. Inclusion Criteria

The Ibermutua workers’ injuries were analyzed, as were their work-related diseases. Every injury at work or "in itinere" affecting any ocular structure was considered as a WREI. Eye injuries were classified by a physician according to Clasificación Internacional de Enfermedades, 9º Revision, Modificación Clínica CIE-9-MC [12] and reclassified later by the research group into the last classification of the World Health Organization (WHO), the International Classification of Diseases (ICD-10) [13]. Table 1 shows the 6 main groups and subgroups into which WREIs were divided.

### 2.3. Statistical Analysis

Qualitative variables were summarized using absolute (n) and relative (%) frequencies. Quantitative variables were summarized using the mean ± standard deviation (SD) or median and interquartile range [IQR] according to their distribution. 

Due to the variance and dispersion of the annual incidence, a negative binomial regression was used to examine the relationships between the injury incidence rates and potential risk factors (sex, age and occupation). We obtained a relative risk (RR) to compare variables and different risk factors. 

For each type of injury, the percentage of incidence was calculated from the number of WREI cases recorded per year. The injury incidence per 100,000 insured workers was also determined according to the number of WREIs per 100,000 workers whom Ibermutua insured. A negative binomial regression was used to calculate an average annual percentage change (2008–2018 (AAPC [%])) in the study of injury incidence evolution over the study period. Time trends were also reported as a cumulative percentage change (CPC) between 2008 and 2018.

The data analysis was performed with IBM SPSS statistics version 21.0 (IBM Corp, USA). Statistical significance was considered when the p-value was lower than the alpha error, which was 5%.

## 3. Results

In total, 11,696,259 insured workers were analyzed in the study period (1,063,296.27 ± 80,065.09 per year). A total of 50,265 WREIs affecting an ocular structure were detected. Table 2 shows the results of each kind of injury.

### 3.1. Demographical Data

Over the 10 years, 0.45% of the insured workers suffered from a WREI with a mean age of 38.62 ± 10.57. Those between 35 and 44 years old are the most affected by WREIs (15,992, 32.0%). There were more WREIs in men (44,445, 89.3%) and workers from the industry sector (18,899, 42.6%). Table 3 shows the details of the WREI for each injury and the demographical group.

### 3.2. Injuries Distribution of WREI by Sex, Age, and Occupation

#### 3.2.1. Sex

Keratitis and conjunctivitis were the most prevalent injuries in both sexes, being the WREI of 84.7% of males and 85.1% of females in the sample. In the analysis of the percentage of incidence, a higher statistically significant incidence of keratitis (p < 0.001) and disorders of globe (p = 0.034) was found in males. Females showed a higher percentage of incidence of conjunctivitis than males (p < 0.001) and other disorders of conjunctiva (p = 0.031). The analysis of injury incidence per 100,000 insured workers showed a higher risk of suffering keratitis, visual disturbances, other disorders of the eye and anexa and visual disturbances in males than in females. On the other hand, females were at more risk of suffering conjunctivitis and other disorders of conjunctiva (Table 4).

#### 3.2.2. Age

There are statistical significance differences between the percentage of incidence of “Keratitis” and “Conjunctivitis” in all the groups when compare to the rest of the injuries. A great variability depending of each injury and age groups was observed in the incidence. Table 4 shows that 16–24 age group had the highest risk of suffering “Conjunctivitis”, 25–34 age group had more risk of developing “Keratitis”, “Other disorders of eye and anexa” and “Disorders of globe”, ≥55 age group had more risk of suffering “Other disorders of conjunctivitis” and “Other injuries”. Finally, 35–44 age group showed the highest risk of suffering “Visual disturbances”. 

#### 3.2.3. Occupation

Table 3 shows the four groups that had a higher number of cases of keratitis and conjunctivitis. There were differences comparing the percentage of incidence of both pathologies’ incidences among all the groups, except for the comparison between industry and services (*p* = 0.100 and *p* = 0.092). Agricultural workers showed higher risk of suffering keratitis, other disorders of conjunctiva, visual disturbances and other injuries when we analyzed injury incidences per 100,000 insured workers (Table 4).

### 3.3. Injuries Incidence Per 100,000 Insured and Evolution over the Study Period

The highest average of injury incidence per 100,000 insured workers over the studied period was observed in the keratitis group (Table 5). Table 5 shows the incidence per 100,000 insured workers of each injury and the evolution over the study period. A decrease in the CPC (%) and AAPC (%) in incidence was found in all injuries except for other disorders of the eye and anexa (+13.9% and +0.2 (95% CI −4.4 to +4.4)) The highest change of CPC (%) was obtained between 2008 and 2009 in the keratitis, conjunctivitis, other disorders of conjunctiva and other injuries groups. The analysis of the total number of injury incidences showed the largest decrease in incidences between both the aforementioned years too.

## 4. Discussion

This study has found that the most prevalent WREIs are keratitis (53.1%) and conjunctivitis (31.6%). The percentage of both injuries is higher than the percentage that Fea et al. obtained in their 2008 study in an urgent care department in Torino, with 44.18% of the eye injuries classified as corneal damage and 10.03% as conjunctivitis [3]. Our results also show differences with the results of the recent study of Zimmerman et al. in Israel [14]. Both studies were carried out in urgent care departments, so they studied the general population as well as workers. That fact and the higher exposition of the anterior segment of the eyeball in workers could justify the differences seen in our study.

The Birmingham Eye Trauma Terminology System (BETT) [15] classifies eye injuries as open-globe (full-thickness wound of the eyeball) and close-globe (no full-thickness wound of the eyeball). In a study carried out in Bosnia–Herzegovina [2], 49.3% of WREIs were close-globe injuries versus 27% in other studies carried out in Helsinki and Palermo [16,17]. A mixed classification between the BETT and ICD-10 has been used in two studies [1,9]. These two studies concluded that in 51.0% and 59.4% of the cases studied there was the presence of a foreign body in the anterior segment of the eyeball.

Coming back to our study, we have observed a decrease over time in the injury incidence of every kind of injury, except in other disorders of the eye and anexa. This decrease was especially high between 2008 and 2009 due to the Global Financial Crisis of 2008, which caused an increase in unemployment in Spain from 9.6% in 2008 to 26.94% in 2013 [18,19]. This increase in unemployment had a direct impact on the number of people insured by Ibermutua, especially in industry and construction, which are the sectors with the highest accident rate. There was a decrease in sickness absences in the youngest age group in construction and with temporary contracts when comparing 2010 and 2006 [20]. Thus, the decrease observed in the WREI incidence between 2008 and 2009 is aligned with the rest of the work-related injuries. 

In all risk factors analyzed (sex, age, and occupation), injuries with a higher number of cases and percentage were the keratitis and conjunctivitis incidences. The higher number of cases in males has been associated by some authors with a higher exposition due to the occupation they have [8,10,14,17,21]. In our study, males have a higher RR and had a higher percentage of keratitis incidences (*p* < 0.001), which comes from the damage of the cornea, which is the most exposed eye structure to risk factors associated with the occupation. Industry workers suffered the highest number of cases of WREIs. This result is in accordance with previous studies [1,8] and it is because some industry workers have more manual tasks. Another reason could be the relationship between sex and occupation. In the last quarter of 2018 in Spain, there were 2.8 times more males than females working in industry [22]. We found also statistically significant differences comparing the two most prevalent injuries among the different occupation groups, except for the comparison between industry and services. These two groups have symmetric behavior in keratitis and conjunctivitis, probably due to the classification of the different occupations. Inside the industry group, not only are people working in manufacturing included but also people working in offices. The work made by this last group is similar in risk to that of the services sector. This fact could also explain the differences between the sectors of industry and services, compared to agriculture and construction.

Keratitis and conjunctivitis presented the highest average injury incidence per 100,000 insured workers. However, the way people suffer these injuries is probably different in each occupation group. For instance, both injuries could be produced by a foreign body on the anterior segment [1,7,8,9,10] in industry or construction, by continual work on the computer in services or even by overexposure to the sun in agriculture.

The highest amount of damage being in the anterior segment of the eyeball supports how important it is to pay special attention to the protection of this segment. According to Pheng Fong and Taok [23], the right use of protective equipment and measures would prevent 60–66% of WREIs, which would make a saving of 37 million dollars in Australia. The anterior segment of the eyeball is easy to protect, and the specific type of protection depends on the occupation. Protective glasses or protective face shields can be the best option for construction and industrial workers. For those who work on a computer, it is very important to improve visual ergonomic measures, to use blue light shields, and instill eye drops frequently. The damage by the sun can be reduced by wearing sunglasses for agricultural workers.

It is important to highlight that the present study gives relevant information in as much as it is the longest and widest, with the highest number of cases in Europe. It gives broad knowledge on the epidemiology of WREIs. However, this strength is also a limitation in as much as we cannot make a detailed study of every case to understand the way they have occurred, the consequences, and the evolutions of the injuries. An exhaustive study with fewer cases would be of interest in the future to know all these details and be able to create specific plans for each kind of injury.

## 5. Conclusions

In WREIs, the cornea and conjunctiva are the structures which suffer more injuries. More prevalent pathologies are keratitis and conjunctivitis, in both sexes, any age group, and any occupation.

## Figures and Tables

**Table 1:** The International Classification of Diseases (ICD-10) groups and subgroups classification of work-related eye injury (WREI) by the WHO.

IDC-10 WHO Codes	IDC-10 WHO Subcodes
H16. Keratitis	H16.0 Corneal ulcer
	H16.9 Keratitis unspecific
	H16.2 Keratoconjunctivitis
	H16.1 Superficial Keratitis without conjunctivitis
	H16.8 Other keratitis
H10. Conjunctivitis	H10.3 Acute conjunctivitis
	H10.9 Unspecific conjunctivitis
	H10.8 Other conjunctivitis
H11. Other disorders of conjunctiva	H11.4Conjunctival hyperemia
	H11.3 Conjunctival hemorrhage
	H11.9 Unspecified disorders
H53. Visual disturbances	H53.1 Subjective visual disorders
	H53.8 Other visual disturbances
H57. Other disorders of eye and anexa	-
H44. Disorders of globe	-

**Table 2:** Work-related eye injuries classified by the ICD-10 codes and subcodes between 2008 and 2018.

ICD-10 WHO Codes	ICD-10 WHO Subcodes	*n*	%
H16. Keratitis		**26,674**	**53.1**
	H16.0 Corneal ulcer	18,841	70.7
	H16.9 Keratitis unspecific	2605	9.8
	H16.2 Keratoconjunctivitis	2092	7.8
	H16.1 Superficial Keratitis without conjunctivitis	532	2
	H16.8 Other keratitis	2604	9.7
H10. Conjunctivitis		**15,906**	**31.6**
	H10.3 Acute conjunctivitis	12,051	75.8
	H10.9 Unspecific conjunctivitis	2826	17.7
	H10.8 Other conjunctivitis	1029	6.5
H11. Other disorders of conjunctiva		**1870**	**3.7**
	H11.4 Conjunctival hyperemia	907	48.5
	H11.3 Conjunctival hemorrhage	642	34.3
	H11.9 Unspecified disorders	321	17.2
H53. Visual disturbances		**1263**	**2.5**
	H53.1 Subjective visual disorders	1222	96.7
	H53.8 Other visual disturbances	41	3.3
H57. Other disorders of eye and anexa		**1028**	**2**
H44.Disorders of globe		**826**	**1.6**
Other injuries *		**2698**	**5.4**
**TOTAL**		**50,265**	**100**

ICD-10 = International Classification of Diseases; WHO = World Health Organization; * Other WREIs that do not belong to the six main codes with International Classification of Diseases (ICD-10) codes: H00; H01; H02; H03; H04; H05; H06; H13; H15; H17; H18; H19; H20; H21; H22; H25; H26; H27; H28; H30; H31; H32; H33; H34; H35; H36; H40; H42; H43; H45; H46; H47; H48; H49; H50; H51; H52; H54; H55; H58; H59 [13].

**Table 3:** Work-related eye injuries by sex, age, and occupation in the 7 groups of injuries.

		H16	H10	H11	H53	H57	H44	Other Injuries	TOTAL
**Sex**	**Male**	**23,869**	**13,763**	**1631**	**1130**	**911**	**774**	**2367**	**44,445**
	%	53.7	31	3.7	2.5	2	1.7	5.3	100
	**Female**	**2562**	**1988**	**228**	**128**	**113**	**72**	**258**	**5349**
	%	47.9	37.2	4.3	2.4	2.1	1.3	4.8	100
**Age**	**16–24**	**2262**	**1503**	**115**	**106**	**83**	**78**	**241**	**4388**
	%	51.5	34.3	2.6	2.4	1.9	1.8	5.5	100
	**25–34**	**7988**	**4789**	**439**	**381**	**328**	**280**	**776**	**14,981**
	%	53.3	32	2.9	2.5	2.2	1.9	5.2	100
	**35–44**	**8539**	**5056**	**570**	**413**	**328**	**255**	**831**	**15,992**
	%	53.4	31.6	3.6	2.6	2.1	1.6	5.2	100
	**45–54**	**5512**	**3172**	**474**	**256**	**192**	**147**	**525**	**10,278**
	%	53.6	30.9	4.6	2.5	1.9	1.4	5.1	100
	**≥55**	**2235**	**1324**	**263**	**106**	**102**	**64**	**296**	**4390**
	%	50.9	30.2	6,0	2.4	2.3	1.5	6.7	100
**Occupation**	**Agriculture**	**976**	**353**	**85**	**55**	**29**	**25**	**101**	**1624**
	%	60.1	21.7	5.2	3.4	1.8	1.5	6.2	100
	**Industry**	**9755**	**6456**	**670**	**515**	**347**	**313**	**843**	**18,899**
	%	51.6	34.2	3.5	2.7	1.8	1.7	4.5	100
	**Construction**	**5843**	**3065**	**346**	**201**	**164**	**201**	**635**	**10,455**
	%	55.9	29.3	3.3	1.9	1.6	1.9	6.1	100
	**Services**	**6789**	**4455**	**535**	**343**	**321**	**164**	**787**	**13,394**
	%	50.7	33.3	4	2.6	2.4	1.2	5.9	100

H16 = keratitis; H10 = conjunctivitis; H11 = other disorders of conjunctiva; H53 = visual disturbances; H57 = other disorders of eye and anexa; H44 = disorders of globe.

**Table 4:** Results from the negative binomial regression about the relative risk of suffering WREI according to sex, age and occupation.

		H16	H10	H11	H53	H57	H44	Other Injuries	All Injuries
**Sex**									
**Male**	**RR**	**Ref.**	**Ref.**	**Ref.**	**Ref.**	**Ref.**	**Ref.**	**Ref.**	**Ref.**
**Female**	**RR**	**0.86**	**1.19**	**1.16**	**0.92**	**0.94**	**0.38**	**1.02**	**0.97**
	95% CI	0.83–0.89	1.14–1.24	1.04–1.19	0.85–0.99	0.87–1.01	0.33–0.42	0.95–1.09	0.97–0.98
**Age**									
**16–24**	**RR**	**Ref.**	**Ref.**	**Ref.**	**Ref.**	**Ref.**	**Ref.**	**Ref.**	**Ref.**
**25–34**	**RR**	**1.03**	**0.96**	**1.09**	**1.05**	**1.21**	**1**	**0.9**	**1**
	95% CI	0.98–1.07	0.91–1.01	0.98–1.20	0.95–1.14	1.10–1.34	0.92–1.11	0.85–1.00	0.99–1.07
**35–44**	**RR**	**1.01**	**0.95**	**1.29**	**1.06**	**1.04**	**0.83**	**0.93**	**0.99**
	95% CI	0.97–1.06	0.91–1.01	1.17–1.42	0.97–1.16	0.94–1.14	0.76–0.91	0.86–1.00	0.99–1.00
**45–54**	**RR**	**1.01**	**0.93**	**1.65**	**1**	**0.92**	**0.72**	**0.92**	**0.99**
	95% CI	0.97–1.06	0.88–0.98	1.50–1.82	0.91–1.11	0.83–1.02	0.66–0.80	0.85–1.00	0.98–0.99
**≥55**	**RR**	**0.96**	**0.91**	**2.1**	**0.94**	**1.16**	**0.73**	**1.21**	**0.986**
	95% CI	0.91–1.01	0.86–0.98	1.89–2.33	0.84–1.06	1.03–1.30	0.65–0.82	1.10–1.32	0.98–0.99
**Occupation**									
**Agriculture**	**RR**	**Ref.**	**Ref.**	**Ref.**	**Ref.**	**Ref.**	**Ref.**	**Ref.**	**Ref.**
**Industry**	**RR**	**0.9**	**1.61**	**0.71**	**0.83**	**1.12**	**1.13**	**0.73**	**1**
	95% CI	0.84–0.95	1.47–1.77	0.64–0.79	0.74–0.93	0.97–1.30	0.98–1.31	0.65–0.81	1.03–1.05
**Construction**	**RR**	**0.95**	**1.31**	**0.67**	**0.57**	**1.01**	**1.31**	**0.93**	**1.01**
	95%CI	0.89–1.01	1.19–1.44	0.59–0.75	0.50–0.64	0.87–1.1	1.13–1.51	0.83–1.04	1.00–1.01
**Services**	**RR**	**0.84**	**1.52**	**0.77**	**0.74**	**1.38**	**0.79**	**0.93**	**1**
	95%CI	0.80–0.90	1.39–1.67	0.69–0.86	0.66–0.84	1.20–1.60	0.68–0.92	0.83–1.04	0.99–1.01

H16 = keratitis; H10 = conjunctivitis; H11 = other disorders of conjunctiva; H53 = visual disturbances; H57 = other disorders of eye and anexa; H44 = disorders of globe. RR = relative risk of suffering an accident relative to injury incidence.

**Table 5:** Average injury incidence per 100,000 insured workers, accumulative average annual percent change and cumulative percentage change in incidence of all injuries over study period (2008–2018)

	H16	H10	H11	H53	H57	H44	Other Injuries	All Injuries
**AII**	**228.1**	**136**	**16**	**10.8**	**8.8**	**7.1**	**23.1**	**429.8**
95% CI	225.3–230.8	133.9–138.1	15.3–16.7	10.2–11.4	8.3–9.3	6.6–7.5	20.4–25.8	426.0–433.5
**AAPC (%)**	**−9.8**	**−12.8**	**−7.1**	**−14.1**	**0.2**	**−6.4**	**−12.1**	**−10.5**
95% CI	−12.5 to −7.0	−17.0 to −8.4	−9.9 to −4.2	−26.4 to +0.3	−4.4 to +4.9	−12.8 to +0.5	−18.3 to −5.4	−13.8 to −7.1
**CPC (%)**	**−64.0**	**−72.9**	**−52.4**	**−9.2**	**13.9**	**−35.6**	**−66.6**	**−65.5**

H16 = keratitis; H10 = conjunctivitis; H11 = other disorders of conjunctiva; H53 = visual disturbances; H57 = other disorders of eye and anexa; H44 = disorders of globe. AII = average injury incidence per 100,000 insured workers. AAPC (%) = average annual percent change (2008–2018). CPC (%) = cumulative percentage change (2008–2018).
